# The impact of cefuroxime prophylaxis on human intestinal microbiota in surgical oncological patients

**DOI:** 10.3389/frmbi.2022.1092771

**Published:** 2023-02-02

**Authors:** Irina Cezara Văcărean-Trandafir, Roxana-Maria Amărandi, Iuliu Cristian Ivanov, Ştefan Iacob, Ana-Maria Muşină, Elena-Roxana Bărgăoanu, Mihail-Gabriel Dimofte

**Affiliations:** ^1^ TRANSCEND Research Centre, Regional Institute of Oncology, Iasi, Romania; ^2^ The Second Surgical Oncology Department, Regional Institute of Oncology, Iasi, Romania; ^3^ “Grigore T. Popa” University of Medicine and Pharmacy, Iasi, Romania; ^4^ Department of Surgery, “Grigore T. Popa” University of Medicine and Pharmacy, Iasi, Romania

**Keywords:** microbiome, metatranscriptomics, antibiotic therapy, bioinformatics, 16S NGS, cefuroxime

## Abstract

**Introduction:**

The intestinal microbiota is vital to human health, and has a profound influence on several biological processes including inflammation and pathogen resistance. Antibiotic intake greatly impacts bacterial diversity, can increase antibiotic resistance and impair the equilibrium between bacterial species. The key to grasping post-antibiotic effects on the gut microbiota rests on the implementation of a suitable procedure to isolate microbial DNA and a meticulous consideration of experimental sequencing artefacts.

**Methods:**

We herein report the bacterial community dynamics of a cohort of 128 surgical oncology patients before and after the intravenous administration of cefuroxime, an antibiotic routinely used in surgical antibioprophylaxis with proven efficiency against both gram-positive and gram-negative bacteria. In our study, we analyzed patient fecal samples collected through rectal examination before and 7 days post cefuroxime treatment by employing a high-throughput sequencing assay which targets the V3–V4 region of the 16S rRNA gene. A first challenge in applying the study design was to extract an appropriate amount of DNA characteristic to the sampled microbiota, which implied the use of both mechanical (ceramic beads) and chemical (proteinase K, lysozyme and lysostaphin) lysis.

**Results:**

Gut microbiota richness and composition was significantly different between the two groups, but most differences were determined by additional perioperative procedures, rather than antibioprophylaxis. Intestinal microbiota composition was not significantly changed one week post cefuroxime treatment when compared to pre-treatment condition for patients without mechanical bowel preparation, but some loss in taxonomic variety could be observed.

**Discussion:**

Taken together, cefuroxime does not promote short-term dysbiosis in surgical patients without any additional perioperative procedures.

## 1 Introduction

The microbiome of the human colon is composed of intricate bacterial communities, fungi, archaea, viruses and eukaryotic parasites, with a structure that is challenging to appropriately evaluate and quantify (Kanangat and Skaljic, 2021; Weiland-Brauer, 2021). By adjusting the host’s immunological, endocrine and neurological pathways, the gut microbiota has a major impact on crucial human processes such as digestion, metabolism and inflammation (Sommer and Backhed, 2013). Approximately 1000 endemic bacterial species populate the human gastrointestinal tract, having a critical role in determining the host’s health or disease state, as well as maintaining microbial-host homeostasis. As a natural barrier of the human body, the colonic mucosa constantly interacts with this bacterial population, being influenced and impacting the equilibrium between a broad range of bacterial species (Sherwin et al., 2018).

Despite variations in richness and complexity among different individuals and across various sites of the gut (Palm et al., 2015), the microbiome ensures a certain level of resilience against external disturbances. Changes in diet and intake of chemicals that can act as antibiotics target specific bacterial populations, producing dysbiotic alterations that are reflected in the overall distribution of bacterial species (Lynch and Pedersen, 2016; Ramirez et al., 2020). Such changes may create a favorable environment for opportunistic bacteria to develop and become predominant, and dietary habit changes have been demonstrated to do so (Sonnenburg and Sonnenburg, 2014; Rinninella et al., 2019). A well-known example of a dramatic consequence of abnormal microbiome equilibrium is *Clostridioides difficile* proliferation and infection, which can be considered a new epidemic generated by antibiotic abuse (Johanesen et al., 2015). Short-term antibiotic therapy may have consequences on the gut microbiota that induce long-term dysbiotic conditions, which can assist the progression and worsen the disease (Vangay et al., 2015). Dysbiosis occurs when intestinal bacterial homeostasis is disrupted and has been linked to numerous illnesses, including type 2 diabetes, obesity, inflammatory bowel disease, asthma, rheumatic disorders, neurodegenerative diseases as well as colorectal cancer (Shreiner et al., 2015). An imbalance in bacterial composition, changes in bacterial metabolic activities, or changes in bacterial distribution within the gut are all symptoms of dysbiosis. Loss of beneficial bacteria, overgrowth of potentially pathogenic bacteria, and loss of overall bacterial diversity are the three types of dysbiosis that coexist frequently (Fujimura et al., 2010; Hsu et al., 2019). Following antibiotic therapy, the dysbiotic phase presents a window of opportunity for disease-causing bacteria to enter the host intestine. The dysbiosis brought on by antibiotic treatment, which is anticipated to cause a loss of stability in the species composition of the gut microbiota and even the extinction of different species, may be the cause of the variation in antibiotic resistance’s effects (Leonidas Cardoso et al., 2020). Thus, understanding the physiology of the gut microbiome and how it is affected by antibiotic stress is a crucial milestone for all of patients, physicians and other personnel working in the medical system. Antibiotic-resistant species are now more than ever in the center of multiple hospital-acquired infections, and the irresponsible prescription of antibiotics is the engine that sets this vicious cycle in motion.

Studies in the role of the microbiome in health and disease have been greatly facilitated by advancements in high-throughput sequencing technologies (Qin et al., 2010; Fraher et al., 2012; Yatsunenko et al., 2012; Proctor et al., 2019).While complex metagenome assembly strategies from shotgun sequencing data are the most accurate in microbiome profiling and novel species discovery and characterization (Gill et al., 2006; Huse et al., 2012; Almeida et al., 2019; Leviatan et al., 2022), microbial profiling by 16S ribosomal RNA (rRNA) gene sequencing is one of the most common methods for studying bacterial phylogeny and taxonomy. Nevertheless, caution must be exercised when choosing the hypervariable regions for sequencing, and appropriate amplicon primer design for 16S rRNA gene sequencing should be employed, as these factors can greatly influence the study’s outcome (Armougom and Raoult, 2009; Wang and Qian, 2009; Jumpstart Consortium Human Microbiome Project Data Generation Working G, 2012; Abellan-Schneyder et al., 2021). One of the most frequently used primer sets for the study of bacterial diversity in different environments is the 341F/785R pair corresponding to the V3-V4 hypervariable regions of the 16S rRNA gene, described by Klindworth et al. (2013). The adequate lysis of heterogeneous communities of microbial cells (both gram-positive and gram-negative) without inducing genome damage is another major challenge (Virgin and Todd, 2011; Human Microbiome Project, 2012; Brooks et al., 2015). To explore the natural microbial community by high-resolution molecular approaches including Next Generation Sequencing (NGS), it is particularly essential to develop a sensitive and reproducible DNA extraction method that facilitates isolation of microbial DNA of sufficient quantity and purity from all the existent microbial species.

The numerous reports on surgical site infections (SSI) as postoperative complications over the past few decades urged to establishing a routine use of preoperative antibiotics in surgical approaches. After demonstrating efficacy in several clinical trials, cephalosporins became the most used drugs for surgical prophylaxis in general surgeries (de Lissovoy et al., 2009; Korol et al., 2013; Carvalho et al., 2017; Bhangu et al., 2018 Halawi et al., 2018; Crader and Varacallo, 2022). Cefuroxime, a second-generation cephalosporin, is efficient against both gram-positive and gram-negative bacteria, and can be administered in combination with other antibiotics if needed. Additionally, it represents a safe and affordable drug, and is the most stable β-lactam antibiotic used to reduce the risk of post operative surgical site infections, sepsis, or abscesses (Geroulanos et al., 2001; Bratzler et al., 2013; Sastry et al., 2022). A literature survey regarding research on the impact of cefuroxime on human microbiomes revealed only a few studies which target the composition of the gut microbiota among newborns whose mothers had received Cefuroxime prior delivery, but provide confounding results (Kamal et al., 2019; Dierikx et al., 2019; Jokela et al., 2022; Bossung et al., 2022). A recent study revealed few overall changes in the bacterial diversity of the mouse gut microbiota 5 days post cefuroxime administration (Hertz et al., 2020), but to our knowledge, no studies have been yet performed to assess the impact of cefuroxime antibioprophylaxis on surgical oncological patients.Of note, preoperative oral antibiotic treatment (cefotetan or cefoxitin - second-generation cephalosporins or cefazolin and metronidazole as a cost-effective alternative) in combination with mechanical bowel preparation (MBP) is recommended in cases of colorectal surgery (Allegranzi et al., 2016) with the aim of decreasing bacterial density and SSI (Poggio, 2013). A recent meta-analysis of 38 randomized clinical trials revealed that the combination of MBP with oral antibiotics (metronidazole, neomycin, kanamycin, kanamycin with erythromycin, neomycin with erythromycin, kanamycin with metronidazole, neomycin with metronidazole, and tobramycin with metronidazole) resulted in the lowest rate of SSI after elective colorectal surgery (Toh et al., 2018), but that the reduction of SSI was not significantly different between the group receiving a combination of MBP and pre-operative antibiotic treatment, and the group receiving pre-operative antibiotic only. The impact of the bowel preparation method itself on gut microbiota composition is still under question, with reports often not reaching consensus due to various reasons including lack of analytical depth (Nagata et al., 2019). A recent report highlights minor shifts in gut microbial composition in non-surgical patients undergoing MBP, but a substantial impact of MBP combined with oral antibiotics (neomycin) in the gut microbiota of surgery patients, with compositional changes persisting in the early postoperative period, with a later repopulation to baseline (Nalluri-Butz et al., 2022).

The main objective of the current study was to assess the human microbiome’s plasticity following antibiotic challenge, as well as its capacity and degree of recovery after surgical intervention. Shifts in gut microbiota composition before (M) and after (T) perioperative antibiotic treatment were comparatively analyzed in a group of 128 surgical oncological patients, by high-throughput sequencing analysis of the V3-V4 region of the 16S rRNA gene in patient stool samples. We must acknowledge the significance of a suitable, gut-friendly preoperative prophylactic antibiotic program given the hypothesis that even short-term antibiotic treatment might lead to long-term dysbiotic conditions.

## 2 Materials and methods

### 2.1 Patients

The study group consisted of 128 (77 females and 52 males aged 30-94 years, average age 62.96, median age 66) surgical oncological patients admitted for noninfectious issues and with no antibiotic treatment in the previous 3 months, which were consecutively sampled from April 2021 to July 2022 at the Regional Institute of Oncology (IRO) Iasi, Romania, and were qualified for prophylactic antibiotic administration. A standard prophylactic antibiotic regimen was given to all patients preoperatively, in accordance to clinical practice guidelines for antimicrobial prophylaxis in surgery (Bratzler et al., 2013): a single dose of 1.5g cefuroxime administered intravenously before incision, to a maximum 2 doses in 12 hours, depending on the duration of the surgery. Patients which already had an ongoing antibiotic treatment were excluded from the cohort to prevent false changes in the microbiome. Paired samples - pretreatment (M) and 7 days after treatment (T) - were collected for the molecular analysis of the microbiome. If mechanical bowel preparation (MBP) was performed in addition to systemic antibioprophylaxis, sample M was collected prior to initiation of MBP. Applied MBP included: polyethyleneglycol (PEG) – 39 patients; enema – 48 patients; no mechanical MBP (NO) – 41 patients.

Inclusion criteria:

- Adult patients with any type of surgery involving antibioprophylaxis, with or without MBP;
- A pause of at least 21 days from neoadjuvant treatment (chemotherapy, radiotherapy), if applicable;
- Signed informed consent for fecal sampling and processing.
- Exclusion criteria:
- Systemic and oral antibiotic therapy in the past 30 days (for infectious pathologies - e.g., urinary tract infections);
- History of mechanical bowel preparation in the past 30 days;
- Ileostomy at the time of admission or if the operating protocol required the formation of an ileostomy;
- Patients requiring surgery without antibioprophylaxis (e.g., breast surgery, plastic surgery);
- Late resumption of intestinal transit (more than 10 days after surgery) or occlusive syndromes.

### 2.2 Sample collection

Two stool samples were collected from each patient: a preoperative sample (M), within the first days of patient hospitalization, before the surgical intervention, MBP (if applicable), and the administration of a single dose of a second-generation cephalosporin, and a postoperative one (T), 7 days after antibiotic administration.

Each sample was collected by rectal examination and spread onto a previously annotated 5x3 cm filter paper (on an area of at least 2 cm^2^), air-dried for at least 30 minutes at room temperature (avoiding positioning the samples in areas with strong air currents), folded in half (with the sample inside), and individually stored in a sealable plastic envelope, specially destined for transport.

After collection, samples were sent to the molecular biology laboratory, refrigerated at 4°C if examined immediately, otherwise stored frozen at -20°C until DNA extraction.

The Institutional Ethics Committee of the Regional Institute of Oncology approved the study protocol and sample size. Written informed consent was obtained from each patient to utilize their fecal samples.

### 2.3 Sample processing and 16S RNA sequencing

#### 2.3.1 DNA extraction

The DNA extraction procedure was optimized and performed in batches of 12 samples to prevent potential cross-contaminations. DNA isolation was performed using the NucleoSpin^®^ Soil kit (Macherey-Nagel, Düren, Germany) according to the manufacturer’s instructions, with a series of additional procedures as follows. The smeared portions of filter paper were cut in thin strips with sterile scissors in order to avoid contamination. The strips from each sample were added to 1.5 mL sterile Eppendorf tubes. The NucleoSpin^®^ Soil protocol was followed using two alternative lysis buffers, SL1 and SL2, with and without the Enhancer SX.

#### 2.3.2 Enzymatic and mechanical lysis

In order to achieve the optimal breakdown of both gram-positive and gram-negative bacteria (Yuan et al., 2012), incubation time and enzymatic concentration were tested for three different enzymes with subsequent addition: proteinase K (Thermo Fisher Scientific, Waltham, Massachusetts, US), lysozyme and lysostaphin (Sigma-Aldrich Co.,US). For obtaining higher DNA concentrations with better absorbance rates measured by spectrophotometry (A260/280 and A260/230 closer to 2) we performed sample lysis optimizations as follows: first, lysis buffer-containing samples were incubated with different concentrations of 10, 20, 30 and 40 μL of proteinase K (20mg/mL ~ 600U/mL) at 65°C for 30 min, and alternatively at 37°C for 30, 60, 120 minutes or overnight. Then, lysed samples containing proteinase K were mixed alternatively with two concentrations of lysozyme and lysostaphin as follows: 10 μL or 50 μL of lysozyme (10mg/mL ~ 40 KU/mL) and 1 μL or 3 μL of lysostaphin (1mg/mL ~ 3 KU/mL), respectively, and incubated at 37°C for 15, 30, 45, or 60 minutes.

Mechanical lysis was performed following the enzymatic lysis, using a FastPrep-24™ homogenizer (MP Biomedicals) at 6 m/sec for 40 sec, on 1 mL of sample added to the MN Bead Tubes type A from the NucleoSpin^®^ Soil kit. To obtain a clear supernatant after fecal material lysis, centrifugation was performed twice for 2 min at 11,000 x g, following the manufacturer’s protocol. After the chemical and mechanical lysis, a volume of approximately 700 μL of the supernatant was transferred to a NucleoSpin^®^ inhibitor removal column for adequate binding, washing and elution steps. A final eluate of 30 μL genomic DNA (gDNA) was obtained by heating the SE elution buffer at 80°C, was quantified and stored at -20°C until use.

#### 2.3.3 Positive and negative controls

Multiple DNA negative extraction controls (4 per run) without fecal matter, but otherwise handled in the same manner as samples, were performed through the entire process, including polymerase chain reaction (PCR) amplification (lack of visible bands) in order to identify possible contaminants. Extractions of a microbial mock community were also performed using known concentrations of different bacterial species mixed in a single Eppendorf tube, in order to evaluate the DNA extraction method efficiency (2-4 per run).

The microbial mock community consisted of defined ratios of cells from 6 human associated bacterial species including Gram-negative bacteria (*Escherichia coli*, *Klebsiella pneumoniae*, *Serratia marcescens*) that are easier to lyse, and Gram-positive bacteria (*Enterococcus faecalis*, *Staphylococcus aureus*, *Staphylococcus epidermidis*) that are more difficult to lyse. Bacterial species were obtained from ATCC or isolated from biological samples and cultivated on Columbia 5% sheep blood agar medium according to manufacturer recommendations. The number of viable cells was estimated by plate counting. The mock community was obtained by mixing between 10^7^ and 10^8^ cells of the 6 bacterial species, and was stored at -80°C until DNA extraction.

#### 2.3.4 Quality and quantification of extracted DNA

The concentration of extracted DNA (absorbance at 260 nm) and its purity (absorbance ratios 260/230 and 260/280) were determined spectrophotometrically using NanoDrop (Thermo Fisher Scientific, Massachusetts, USA). Ratios from 1.8 to 2.0 were suggestive for lack of protein contamination (Wilfinger et al., 1997). The ratio between absorbance at 260 and 230 nm was used to determine contamination with organic compounds, phenols and carbohydrates. The integrity and size of the DNA samples were assessed by gel electrophoresis on a 2% agarose gel (w/v) stained with ethidium bromide and run in 1x TAE buffer at 180 V.

#### 2.3.5 16S rDNA sequencing

To determine the bacterial composition of each sample, a 16S metagenomic sequencing library was generated, according to Illumina’s instructions (Illumina, 2013). Briefly, we targeted the V3-V4 region of the 16S rRNA gene throughout a first PCR using the following specific primer pair sequences with overhang adapters F: 5’-TCGTCGGCAGCGTCAGATGTGTATAAGAGACAGCCTACGGGNGGCWGCAG-3’ and R: 5’-GTCTCGTGGGCTCGGAGATGTGTATAAGAGACAGGACTACHVGGGTATCTAATCC-3’ that yielded an amplicon of approximately ~460 bp (Klindworth et al., 2013) in 280 samples (256 paired patient samples, 2 sample duplicates, 12 negative controls and 10 positive controls). We employed 2.5 μL microbial genomic DNA and 5 μL of the each forward and reverse 1 μM primers, combined in a 12.5 μL 2X KAPA HiFi HotStart Ready Mix (Roche Holding AG, Basel, Switzerland) in a final volume of 25 μL with the following thermal cycling conditions: denaturation at 95°C for 3 min, followed by 25 cycles at 95°C, 55°C and 72°C for 30 s each, and a final extension of 5 min at 72°C to confirm full amplification.

The resulting sequences were then purified with Agencourt AMPure XP magnetic beads (Beckman coulter, Brea, US) to clean the 16S V3-V4 amplicons away from free primers and primer dimer species. For this step, we implemented an automatic purification program with the employment of the BIOMEK^®^ FXP workstation (Beckman coulter, Brea, US) following the reaction cleanup protocol, in order to generate high-quality results. Subsequently, a second PCR was performed from 5 μL of the purified PCR amplicons to attach the dual indexes and Illumina sequencing adapters, using the Nextera XT Index Kit V2 set A (Illumina, San Diego, USA) with 5 μL Nextera XT Index 1 Primers F (N7XX) (10 M) and Nextera XT Index 2 Primers R (S5XX) (10 M) each, 25 μL of 2X KAPA HiFi HotStart Ready Mix (Roche Holding AG, Basel, Switzerland) and 10 μL ddH2O in a final reaction volume of 50 μL. The thermal cycling program was accomplished after a 3 min activation and denaturation at 95°C, followed by 8 cycles at 95°C, 55°C and 72°C for 30 s each, and a final elongation step of 5 min at 72°C.

Following a second purification with AMPure XP XP magnetic beads (Beckman coulter, Brea, US) to clean up the final library before quantification, the PCR products DNA concentration was evaluated using a Qubit 4 fluorometer and the Qubit™ 1X dsDNA High Sensitivity (HS) Assay Kit (Thermo Fisher Scientific, Massachusetts, USA). The barcoded amplicon libraries were pooled in equimolar concentrations to generate a 4 nM library. The pool of samples was then denatured to a final concentration of 12 pM and combined with 20% Phix control (Illumina, San Diego, USA). The V3-V4 region of the 16S rRNA gene libraries were sequenced using a MiSeq Reagent Kit (Illumina, San Diego, USA) on the Illumina MiSeq platform with the 300 paired-end (2 × 300 bp (PE300)) sequencing protocol.

### 2.4 Bioinformatics and statistical analysis

#### 2.4.1 Taxonomic profiling

Demultiplexed sequences were processed using the dada2 package v 1.22 (Callahan et al., 2016a) implemented in R (version 4.1.2), following the DADA2 pipeline (Callahan et al., 2016b). In brief, primers were trimmed and sequences containing ambiguous bases were removed. After inspecting quality profiles, forward and reverse reads were truncated at 255 and 215 bases, respectively. This ensured an overlap of 20 bases, when taking into account trimmed primers and an average amplicon length of 467 bases, as expected for the V3-V4 region. Different filtering strategies were tested in order to retain an adequate number of sequences for merging, and the parameters maxEE=6 and truncQ=2 were finally selected for the dataset. Forward and reverse reads were subsequently merged, and chimeras were detected and removed. Merged sequences with fewer than 350 bases were removed. Taxonomy was assigned using dada2’s implementation of the RDP Naive Bayesian Classifier (Wang et al., 2007) (80% confidence), trained on the V3-V4 region of sequences from the SILVA database (release 132) (Callahan, 2018), followed by species-level assignment by exact matching. ASVs which were classified as eukaryotic, mitochondrial or chloroplast, as well as ASVs unclassified at phylum level were removed from further analyses. Samples which retained fewer than 5000 reads after taxonomic filtering were discarded, as well as corresponding samples from the other group in order to maintain data pairing. A total of 120 paired samples from each group (M or T) remained. Out of the T group, 38 samples had no pre-operative bowel cleansing (NO group), 46 had pre-operative enema (Enema group) and 36 had PEG MBP (PEG group).

Phyloseq objects which included ASV tables, taxonomic classifications and metadata were generated with the phyloseq package v 1.38 (McMurdie and Holmes, 2013). After merging species-level unclassified sequences, ASVs which were present in less than 1% samples were removed. A neighbor-joining phylogenetic tree was constructed using the phangorn package v 2.9.0 (Schliep, 2011), starting from a multiple sequence alignment of ASVs performed with the DECIPHER package v 2.22 (Wright, 2016), fitted with a GTR+G+I model.

#### 2.4.2 Alpha and beta diversity

Non-phylogenetic alpha diversity indices were computed using phyloseq v 1.38 (McMurdie and Holmes, 2013), while Faith’s phylogenetic distance was calculated using picante v 1.8.2 (Kembel et al., 2010). Statistical significance was assessed using the Wilcoxon signed-rank test (paired samples).

The Bray-Curtis sample-wise distance matrix was calculated in phyloseq from count data normalized through variance stabilizing transformation (VST), as implemented in the DESeq2 package (Love et al., 2014). UniFrac distances were calculated from raw count data using GUniFrac package v 1.6 (Chen et al., 2012).

Permutational multivariate analysis of variance (PERMANOVA) and analysis of similarities (ANOSIM) were performed on the Bray-Curtis distance matrix and variance adjusted weighted UniFrac distances in order to estimate significance in compositional dissimilarity. PERMDISP was performed on the same metrics for assessing intra-group dispersion homogeneity using the vegan package v 2.6-2 (Oksanen et al., 2022). Pairwise permutation MANOVA on variance-adjusted weighted UniFrac distances was performed using the RVAideMemoire package v 0.9-81-2 (Hervé, 2022). Multiple testing P value adjustment was performed using the Benjamini & Hochberg false discovery rate (FDR) method, with FDR < 0.05 being considered significant.

Dimensional reduction through principal coordinates analysis (PCoA) based on the Bray-Curtis dissimilarity matrix and variance adjusted weighted UniFrac distances was performed with the phyloseq package in order to visualize microbiota similarity and grouping across samples. All plots were generated using ggplot2 v 3.3.6 (Wickham, 2016).

#### 2.4.3 Differential abundance analysis

Significance in differential abundance between groups was assessed through four different methods, as per recent recommendations regarding choice of differential abundance methods (Nearing et al., 2022), from genus-agglomerated phyloseq objects. Linear discriminant analysis effect size (LEfSe) was performed on untransformed data, with an LDA cutoff of 4, as implemented in the microbiomeMarker package v 1.0.2 (Cao et al., 2022). ANOVA-Like Differential Expression was performed using the ALDEx2 package v 1.26.0 (Fernandes et al., 2014), using center-log transformed data. Analysis of compositions of microbiomes with bias correction was performed using the ANCOMBC package v 1.4.0 (Lin and Peddada, 2020). Lastly, edgeR v 3.36.0 (Robinson et al., 2010) was used to normalize raw data into trimmed mean of M-values (TMM) counts, which were then used as input for variance modeling at observational level (voom), as implemented in limma v 3.50.3 (Ritchie et al., 2015).

The genus Escherichia/Shigella was referred to as Escherichia, considering that all subjects had no symptoms of Shigella infection at the time of sampling.

## 3 Results

### 3.1 Sample lysis optimization and DNA concentration

Enzymatic lysis was optimized to overnight incubation at 37°C with 1 mL SL2 (sample lysis 2 from the NucleoSpin^®^ Soil kit) and 40 μL proteinase K, followed by 1 h incubation at 37°C with 50 μL lysozyme (10mg/mL ~ 40 KU/mL) and 3 μL lysostaphin (1mg/mL ~ 3 KU/mL). After each reagent addition, the samples were vortexed vigorously for a few minutes.

These adjustments allowed us to achieve higher DNA concentrations and purity with absorbance rates varying between 1.77 and 1.99 for A260/280 (average: 1.91) and 1.99 to 2.15 for A260/230 (average: 2.01) in the M group and 1.75 and 2 for A260/280 (average 1.87) and 1.98 to 2.19 for A260/230 (average 2.11) in the T group. The DNA concentration in the M samples ranged from 285.9 ng/μl to 28.7 ng/μl (average: 50.96 ng/μl) and in the T samples varied between 192.6 ng/μl to 28.3 ng/μl (average: 49.71 ng/μl).

### 3.2 16S rDNA sequence reads processing

A total of 43,041,359 reads from 3 runs were processed from 280 input fastq files: 256 paired samples, 2 sample duplicates, 12 negative and 10 positive controls. Sample sequencing depth ranged from 3,283 to 3,681,248 reads per sample (average 153,719 reads, median 78,824 reads). After all processing steps before taxonomy assignment, a total of 28,619,858 reads remained, corresponding to 66.49% of initial reads, with 493 to 2,549,573 merged non-chimeric reads per sample (average 102,213, median 52,612). The final amplicon sequence variant (ASV) table contained 15,509 unique ASVs for all 280 samples, including controls and sample duplicates. After taxonomic assignment, filtering and removing samples with fewer than 5000 reads, as well as negative and positive controls, 13,795 unique ASVs remained in 240 samples (120 in each group), from which 720 could be classified at species level. Since multiple unique ASVs could be classified as the same species or were left unclassified at species level, sequences were merged at species-level in order to remove spurious ASVs. This resulted in a total of 666 unique ASVs in 240 samples (120 samples from each group), 635 shared between M (before antibiotic treatment) and T (7 days post-antibiotic treatment) groups, 17 unique for the M group, and 14 unique for the T group (**Supplementary Tables 1, 2**).

Negative controls contained an average of 309 reads after all filtering steps, which were classified at genus level to a total of 60 ASVs, with up to 29 different genera within a single negative sample (mean 9, SD = 8). No genera were present in all negative controls, but the genus *Escherichia* could be identified in 10 out of 12 negative controls, with 16.8% of negative control reads being classified in this genus. However, absolute counts for this genus were very low (mean 52, SD = 34). The genus *Bacteroides* was present in 7 out of 12 negative controls (18.41% of reads), while the genus *Prevotella* could be identified in 4 out of 12 negative controls (13.64% of reads). The other 57 genera were present in less than 3.5% relative abundance. On average, the relative abundances of *Escherichia*, *Bacteroides* and *Prevotella* in all patient samples were 4.51%, 13.77% and 4.63%, respectively (SD = 8.52%, 9.69% and 7.35%, respectively). Considering the low overall absolute counts in negative controls, as well as the expected microbiota composition in human stool samples (Thomas et al., 2011), taxa identified in negative controls were not removed from samples.

Mock community analysis from three types of positive controls with defined community structure containing both gram-negative and gram-positive bacteria (CP1, CP2 and CP3) revealed some variation from expected relative abundances, with the highest bias in variation for gram-negative bacteria *Klebsiella pneumoniae* (**Supplementary Figure 1**). Overall, a mean shift of 5.93% from expected relative abundances was observed (except *Klebsiella*, with 18.65% positive shift), with a mean underrepresentation of gram-positive bacteria, which are more difficult to lyse, of 6.53%.

For CP1, mean obtained relative abundances were: 43.65% *Klebsiella*, 20.53% *Enterococcus*, 21.66% *Escherichia* and 14.16% *Staphylococcus* (expected 25% of each genus). For CP3, mean obtained relative abundances were 50.76% *Serratia*, 23.88% *Staphylococcus*, 18.38% *Enterococcus* and 6.96% *Escherichia* (expected 45% *Serratia*, 25% *Staphylococcus, 25*% *Enterococcus* and 5% *Escherichia*). Finally, in the case of CP2, we obtained mean relative abundances of 52.89% *Escherichia*, 31.73% *Staphylococcus* and 15.37% *Enterococcus* (expected 50% *Escherichia*, 25% *Staphylococcus* and 25% *Enterococcus*). This suggests that while bias towards gram-negative bacteria is observed, the DNA extraction protocol worked well for both gram-positive and gram-negative bacteria, since all control bacteria could be correctly identified at genus level.

### 3.3 Bacterial diversity

**Figure 1:**
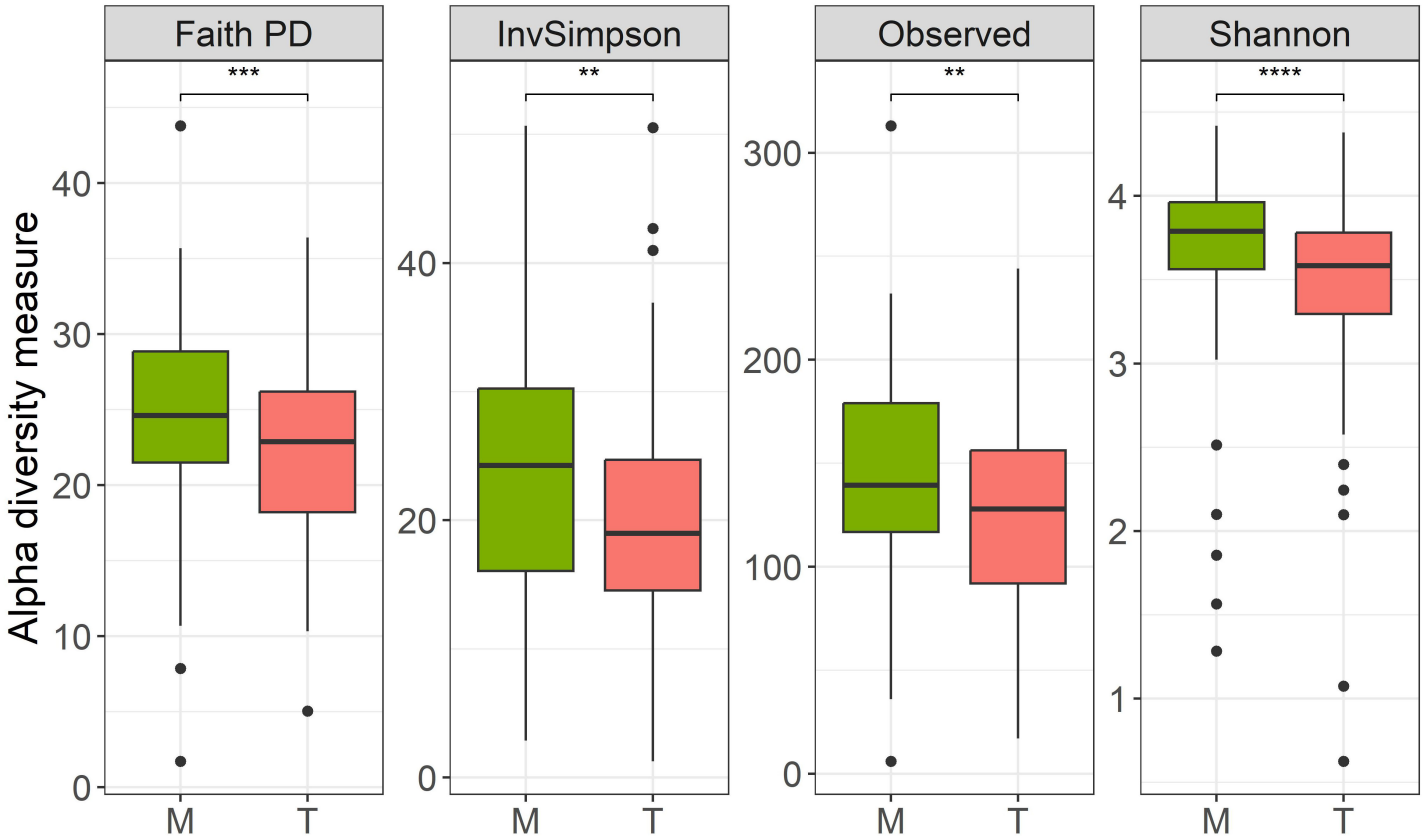
Alpha diversity measures (Faith’s Phylogenetical distance, Inverse Simpson’s index, Observed ASVs and Shannon Diversity index), before (M)/7-days post antibiotic treatment (T); paired Wilcoxon test: **p-value <0.01, ***p-value < 0.001, ****p-value < 0.0001.

Overall, inter-group diversity was found to be statistically different, as indicated by measured alpha diversity indices (**Figure 1**), with the T group (7 days post-antibiotic treatment) being less diverse than the M group (before treatment), suggesting that antibiotic treatment had an impact on species richness and that not all initial gut microbiota had repopulated after antibiotic treatment.

**Figure 2:**
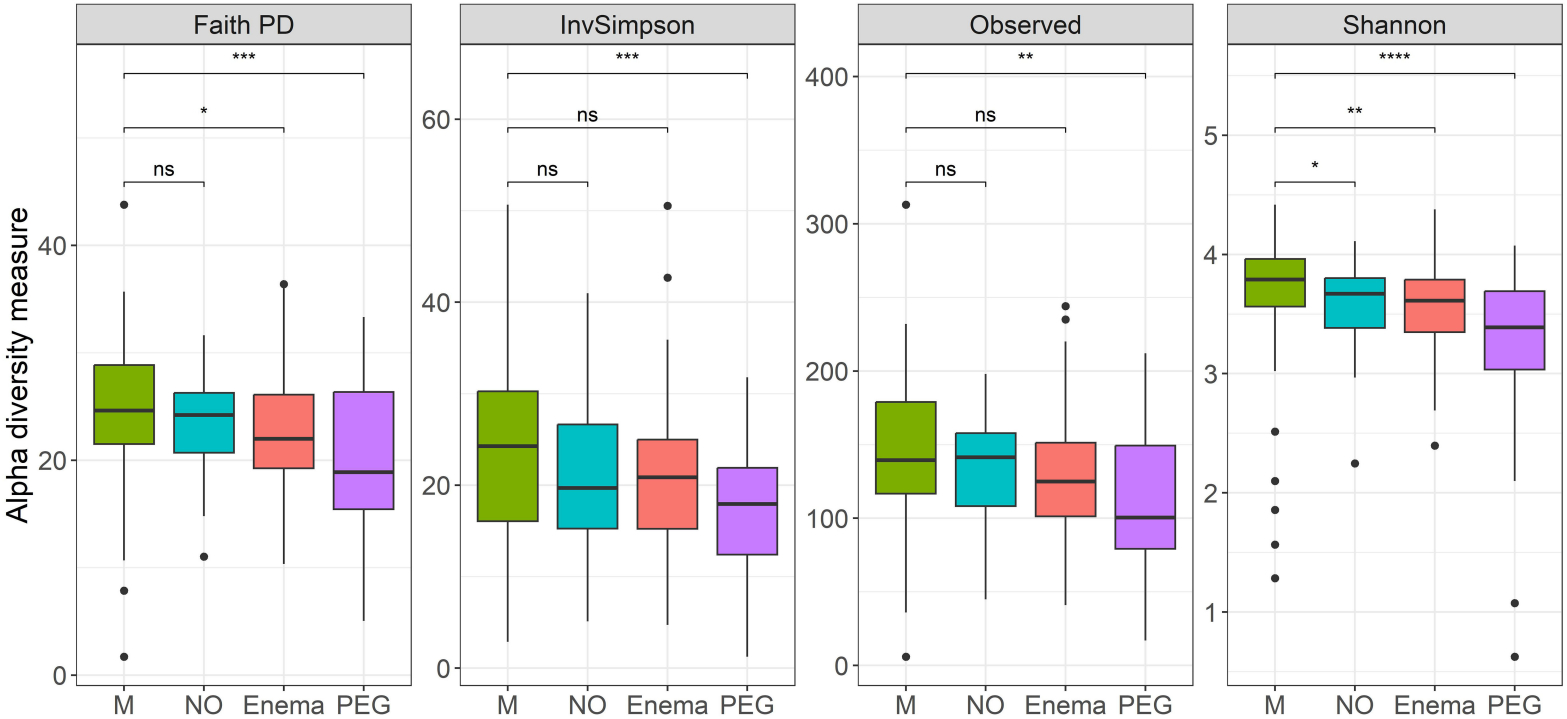
Alpha diversity measures (Faith’s Phylogenetical distance, Inverse Simpson’s index, Observed ASVs and Shannon Diversity index), before (M)/7-days post antibiotic treatment, when considering MBP method: no bowel cleansing (NO); with preoperative enema (Enema); with preoperative PEG MBP (PEG); paired Wilcoxon test: **p-value < 0.01, ***p-value < 0.001, ****p-value < 0.0001.

However, if preoperative bowel cleansing method is also taken into account, a clear distinction in terms of alpha diversity can be observed between groups (**Figure 2**), with significant differences found in all measured metrics between before-after antibiotic treatment in patients undergoing preoperative MBP with polyethyleneglycol (PEG).

Less significant differences were found between before-after antibiotic treatment in patients with preoperative enema in terms of Faith’s phylogenetical distance and Shannon index, as well as before-after antibiotic treatment in patients without any preoperative preparation in terms of Shannon index. This suggests that regardless of preoperative preparation, some loss in species richness occurred after antibiotic treatment.

### 3.4 Bacterial composition

**Figure 3:**
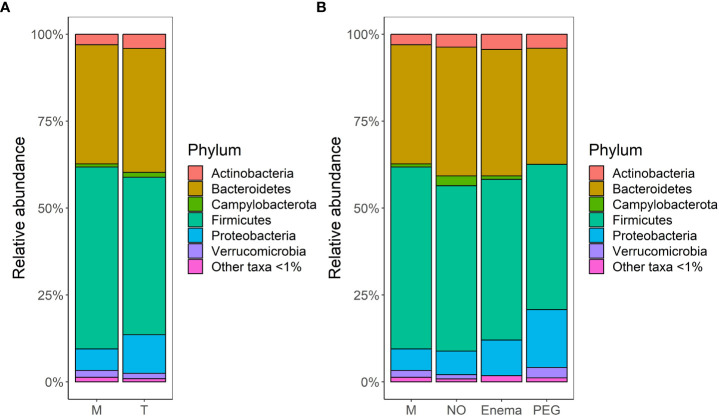
Bacterial community structure merged at phylum level per each group, in terms of relative abundances: **(A)** before (M)/7-days post antibiotic treatment (T); **(B)** before (M)/7-days post antibiotic treatment, when considering MBP method: no bowel cleansing (NO); with preoperative enema (Enema); with preoperative PEG MBP (PEG); Phyla with lower than 1% relative abundance are grouped together under ‘Other taxa <1%’.

The average bacterial composition at phylum level in the M group, according to relative abundances, is 52.3% *Firmicutes*, 34.3% *Bacteroidetes*, with a smaller representation of *Actionobacteria* (3%), *Proteobacteria* (6.2%), *Verrucomicrobia* (2%) and other taxa (**Figure 3A**), as would be expected in the human gut (Stavrou and Kotzampassi, 2017). The T group is less abundant in *Firmicutes* taxa (45.3%), but more abundant in *Proteobacteria* (11%) and *Campylobacterota* (1.4%), suggesting that bacterial composition is indeed impacted by antibiotic treatment. However, when taking into account the preoperative bowel cleansing preparation in the case of gastrointestinal surgical interventions, a bias in compositional shift could be observed for samples from patients which had undergone preoperative PEG MBP (**Figure 3B**). As such, patients without any MBP had comparable relative abundances in all major phyla both pre-treatment (M group) and 7 days post-treatment (NO group), suggesting that pre-operative MBP impacts bacterial composition in addition to antibiotic treatment.

**Figure 4:**
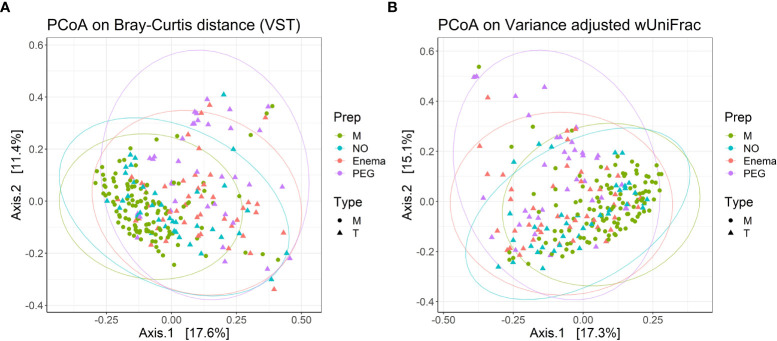
PCoA plots with **(A)** Bray-Curtis dissimilarity from VST-transformed data and **(B)** variance-adjusted weighted UniFrac distance, illustrating distances between communities in individual samples (n=240). Point shapes according to treatment type: circles – samples before antibiotic treatment (M); triangles - samples 7 days post-antibiotic treatment (T); Color according to bowel cleansing preparation: green – samples before antibiotic treatment, with no bowel cleansing (M); blue – samples 7 days post-antibiotic treatment, with no bowel cleansing (NO); red – 7 days post-antibiotic treatment, with preoperative enema (Enema); purple – samples 7 days post-antibiotic treatment, with preoperative PEG MBP (PEG); Ellipse drawn at 95% confidence level. Percentage of variation explained by the first two dimensions are indicated on respective axes.

PCoA analysis using Bray-Curtis dissimilarity and variance-adjusted weighted UniFrac distances showed that both groups (M and T) present high inter-sample variations, with no clear segregation, although M type samples cluster together more readily, suggesting that the M group has a more homogenous composition than the T group (**Figures 4A, B**). Intra-group dispersion was significantly different between M and T groups when using Bray-Curtis dissimilarity (P(perm)=0.0001), but not significant when using variance-adjusted weighted UniFrac distances (P(perm)=0.068). A larger dispersion in the T group suggests that samples which have received cefuroxime treatment tend to have a more individualized community composition than samples which have not.

When taking into account preoperative bowel cleansing preparation in terms of Bray-Curtis dissimilarity, the group receiving PEG MBP was the most dissimilar in terms of dispersion (P(perm)=0.0001/0.0029/0.0149 for PEG vs. M/NO/Enema groups, respectively), while the dispersion for the treatment group without any MBP was not significantly different from the before antibiotic treatment group (P(perm)=0.306) or treatment group with pre-operative enema (P(perm)=0.268). When using variance-adjusted weighted UniFrac distances, no significant differences in group dispersions was observed. Thus, PERMANOVA on variance-adjusted weighted UniFrac distances was used in order to assess significance in group centroid differences.

PERMANOVA suggests that composition differs between M and T groups (R^2^ = 0.03719, P=0.0001), but that only 3.71% of compositional variation between samples can be explained by antibiotic treatment, with an additional 6.13% being explained by pre-operative MBP method (R^2^ = 0.06137, P=0.0001). Analysis of similarities confirmed the weak overall difference in bacterial composition between M and T groups (P=0.0001, R=0.109), as well as the greater impact of pre-operative MBP method on bacterial composition (P=0.0001, R=0.158). Pairwise permutation MANOVAs on variance-adjusted weighted UniFrac distances revealed significant differences in centroid positioning between pre-treatment and all post-treatment conditions (P=0.0039/0.0003/0.0003 for M vs. NO/Enema/PEG, respectively), suggesting that regardless of the stronger effect of the pre-operative preparatory MBP, antibiotic treatment still impacts bacterial composition to some extent. At the same time, distance centroids between the Enema group and the group receiving no MBP were not significantly different (P=0.0594), suggesting that PEG MBP has the most impact on post-antibiotic treatment bacterial composition.

Hierarchical cluster analysis using Bray-Curtis dissimilarity and Ward’s clustering algorithm for VST-normalized abundances revealed that 27 samples from the T group were the most similar to corresponding M samples, clustering together (**Supplementary Figure 2**). Thus, in 22.5% cases, the gut microbiota regained pre-treatment status 7 days after antibiotic treatment. If preoperative MBP is also taken into account, microbiota repopulation occurred for 42.1% patients with no MBP, 19.5% patients with pre-operative Enema and only 5.55% of patients with PEG MBP.

### 3.5 Differential abundance analysis

**Figure 5:**
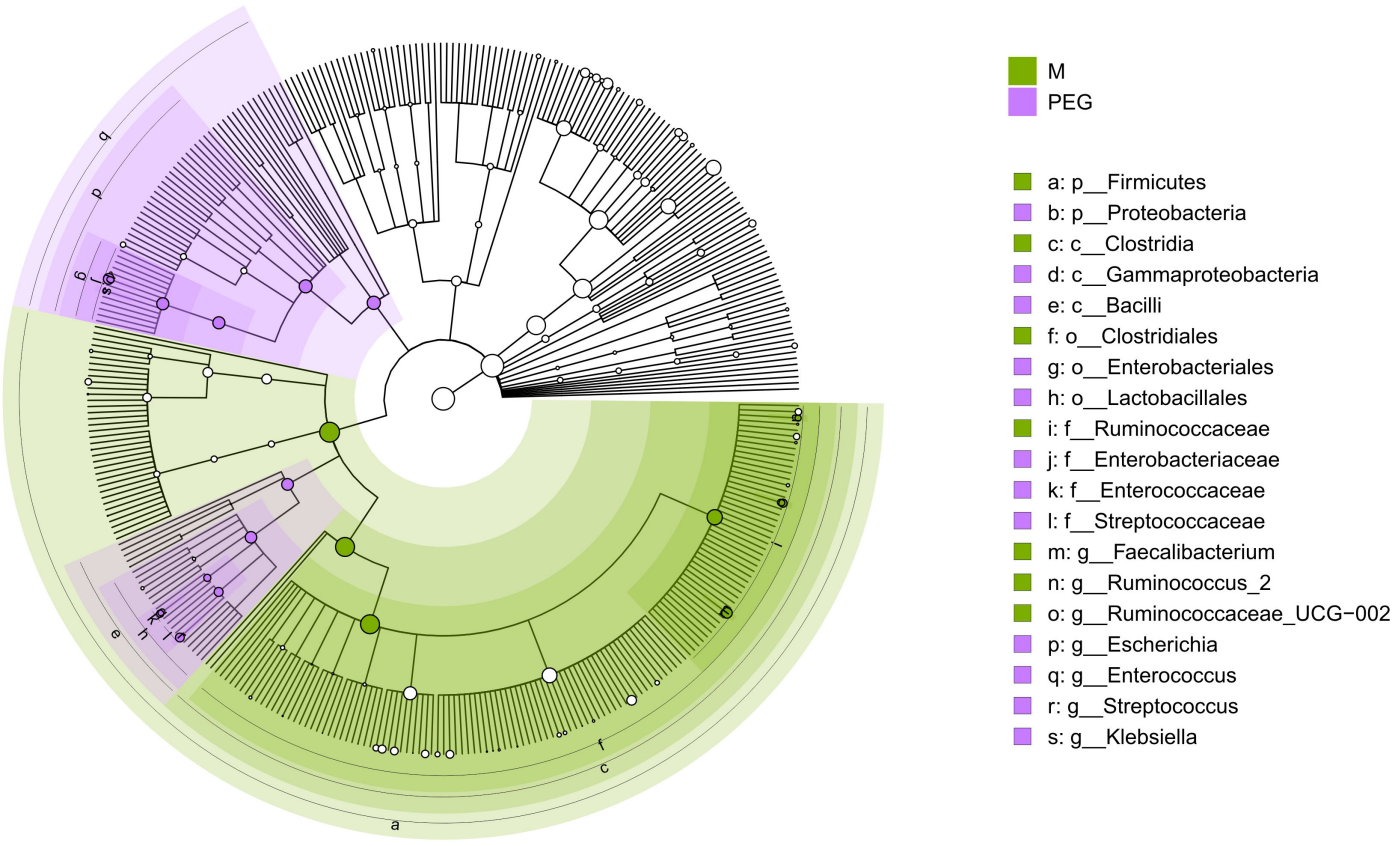
Circular phylogenetic tree (cladogram) showing the phylogenetic distribution of taxa which differ significantly between M and PEG groups, according to consensus between the applied Differential Abundance Analysis methods; green – taxa which are more abundant in samples of the M group; purple – taxa which are more abundant in samples after antibiotic treatment, with preoperative PEG MBP (PEG). Order of hierarchy (from center): Kingdom, Phylum, Class, Order, Family, Genus. Node size is proportional to relative abundance. In the legend, the letter in front of the taxon represents the taxonomy level: p, phylum; c, class; o, order; f, family; g, genus.

Differential abundance analysis allowed for the identification of taxa for which abundance differed significantly between groups. After clustering at genus level, the phyloseq object which was subjected to analysis contained 359 unique genera. LEfSe identified 12 genera differentially abundant between M and T groups, ALDEx2 identified 65, ANCOMBC identified 85, while limma voom identified 106. Notably, intersection of genera identified by all four methods resulted in 7 differentially abundant genera between M and T, which could be further discriminated in the T group by the applied preoperative bowel cleansing method (**Figure 5**).

As such, commensal genera *Faecalibacterium*, *Ruminococcus_2* and *Ruminococcae_UCG-002* were more abundant in samples before antibiotic treatment, suggesting that not all microbiota had regenerated after antibiotic treatment, regardless of applied perioperative preparation methods. At the same time, opportunistic genera including *Escherichia*, *Enterococcus*, *Streptococcus* and *Klebsiella* were more likely to be found in the gut microbiome of patients undergoing PEG MBP.

Of note, pairwise comparisons between preparation type groups using limma voom identified only two differentially abundant taxa between samples before treatment (M) and samples 7 days post-treatment from patients with no bowel preparation (NO), which were also identified as differentially abundant by ALDEx2 and ANCOMBC between M and T groups, but not LEfSe: *Ruminococcaceae_UCG-014* and *Lachnospiraceae_UCG-001*, suggesting that M and NO groups are the least different in terms of composition.

## 4 Discussion

High-throughput sequencing techniques such as 16S ribosomal ribonucleic acid (rRNA) gene amplicon sequencing are frequently used to examine gut microbiota composition. As metagenomic analyses are known to be significantly impacted by DNA extraction (Zhang et al., 2015; Greathouse et al., 2019), 16S rRNA gene sequencing needs to be used meticulously and should involve the careful evaluation of experimental artefacts.

DNA recovery has proven to be a recurring issue in studies involving microbial data collection (Claassen et al., 2013; Guo and Zhang, 2013; Ferrand et al., 2014; Rubin et al., 2014). The effectiveness of cell lysis, as opposed to DNA recovery, has a greater impact on the reviewed microbial composition and gram-positive bacteria are more resistant to lysis than gram-negative bacteria, which often translates to poor representation in relative abundance data (Salonen et al., 2010). Although underrepresentation of gram-positive bacteria occurred in our investigation, suggesting that further optimization is still needed for proper stool sample microbiota characterization, we observed that extraction procedures involving a bead beating mechanical lysis and an enzymatic lysis step produced noticeably better illustrations of the bacterial community structure than procedures omitting either of these phases. Thus, a more complete and uniform profile of the microbial diversity is provided by an increased lysis efficiency. A combination of lytic enzymes (proteinase K + lysozyme and lysostaphin) provided the best depiction of microbial diversity for all samples, most likely due to variations in peptidoglycan structure across bacterial species as highlighted by a recent study (Bergsten et al., 2020).

However, our results outline the need for further DNA extraction protocol optimization in order to more accurately characterize the microbial community from patient stool samples, and other bacteriolytic enzymes, such as mutanolysin, as well as chemical lysis aided by antimicrobial peptides (AMPs), are currently being considered by our group for future DNA extraction protocol optimization.

It must be noted that the capacity of various DNA extraction techniques to accurately represent the microbial diversity in samples cannot be assessed without the use of a control community with an established composition, also called a “mock community”, in order to prevent the preferential isolation of certain bacterial species. This has been continuously stressed in various metagenomic studies, emphasizing the importance of using proper control communities for correctly characterizing the investigated microbiome (Brooks et al., 2015; Fouhy et al., 2016; Kioroglou et al., 2019). The use of *in house* bacterial mock communities, such as we have done in our study, is often encouraged because it can reflect the variability of interesting or relevant taxa more accurately than commercially available communities (Han et al., 2020). However, its comparison to other studies employing commercially available standardized mock communities is limited, and the employment of such communities as additional controls will be considered in future microbiome research studies conducted by our group. Of note, our results show that the DNA extraction protocol worked well for both gram-positive and gram-negative bacteria, even though bias towards gram-negative bacteria was observed. However, as the DNA extraction protocol contributed to gram-negative bacteria overrepresentation, this could impact further result interpretation, especially if only gram-negative bacteria are found as differentially abundant in certain study groups.As microbial resistance is increasing due to antibiotic misuse, an appropriate antibioprophylactic regimen in surgical patients is becoming more challenging. Using 16S amplicon sequencing and principal coordinate analysis (PCoA) on unweighted UniFrac distances to characterize the gut microbiota composition, researchers discovered that antibiotic treatment has a significant impact on the composition of the microbiota and that the dispersion in microbiota composition sometimes increases after antibiotics treatment (Lauka et al., 2021; Guo et al., 2022; Jiao et al., 2022). Our results support these findings, as we found significant intra-group dispersion differences between before (M) and 7 days post-antibiotic treatment (T) groups. In particular, our study found that three genera are most likely to still be depleted 7 days post-cefuroxime treatment, namely *Faecalibacterium*, *Ruminococcus_2* and *Ruminococcae_UCG-002*. All three are gram-positive and belong to the *Firmucutes* phylum, *Clostridia* class and *Ruminococcaceae* family, and *Faecalibacterium* in particular has been shown to promote short fatty acid chain production which can influence intestinal homeostasis through anti-inflammatory cytokine production increase and pro-inflammatory cytokine production decrease (Martin et al., 2017). In addition, species belonging to the genus *Ruminococcus* seem to be consistently present in the healthy human gut, suggesting that they play a significant role in maintaining a normal environment in the gut (Park, 2018), and as such, it is not surprising that they are impacted to a certain extent by antibiotic administration. Cefuroxime is also known to cause lower rates of sensitivity in cases of *Eneterobactericeae* and *Streptococcus*-induced peritonitis (Groteluschen et al., 2020). Our results show that cefuroxime administration permits the repopulation of gut microbiota 7 days post-antibiotic treatment, but only in cases where patients did not undergo a perioperative mechanical bowel preparative procedure. It has been previously reported that bowel cleansing can induce temporary changes in the gut microbial composition, particularly in the relative abundance of *Proteobacteria* (Gorkiewicz et al., 2013), which corroborated with a loss in overall species richness, can suggest a more efficient repopulation of depleted niches by bacteria of this phylum. Indeed, a recent study showed a significant increase in *Proteobacteria* and decrease in *Firmicutes* immediately after colon cleansing with PEG, but recovery of the microbiota to resemble pre-MBP condition one month after MBP (Drago et al., 2016). As such, the observed increase in *Proteobacteria* for our samples could indicate that the colon has not yet had the time to repopulate to its pre-MBP state, and not necessarily an infection caused by the identified differentially abundant opportunistic bacteria. However, the lack of data regarding later post-intervention timepoints urges for future investigations in order to determine if the identified differentially abundant taxa indeed cause SSI or are outcompeted by commensal bacteria in time.

Several issues of the current study could be better addressed in future investigations. A recent report highlights alterations in stool microbiome upon sample freezing without the addition of cryoprotectants (Bilinski et al., 2022). In our analysis, we only report data from both fresh and frozen stool samples, and do not account for potential changes caused by cryopreservation. Other reports highlight the substantial influence of diet on the gut microbiota (Allin et al., 2015), and our study does not account for altered diets between patients, nor before and after surgical intervention. In addition, our study does not account for potential gut dysbiosis induced by diabetes (Sharma and Tripathi, 2019) or obesity (Gao et al., 2018), since metadata regarding body mass index or diabetes status was not collected.

Nevertheless, our results suggest that the gut microbiota is repopulated close to pre-treatment condition 7 days post cefuroxime antibioprophylaxis, and the extent of repopulation greatly depends on other perioperative procedures such as MBP.

## 5 Conclusions

Antibiotics have significant and occasionally long-lasting impact on the intestinal microbiota, inducing a decrease in the quantity of commensals that are beneficial and an increase in commensals that could become harmful. The use of probiotics and antibiotic treatment can be tailored to reduce this “collateral damage” if these effects are better understood.

The choice of DNA extraction technique plays a crucial role in the observed microbial diversity in microbiome studies. Employing a protocol that involves both mechanical and chemical lysis will lead to obtaining utmost species diversity and abundance in all samples.

The added benefit of a metagenomic approach to gut microbiome analysis is that it not only characterizes the microbial community, but also sheds light on its possible physiological effects on the human host. Additionally, 16S rDNA sequencing allows the detection of dysbiosis events in samples, as well as measurement of the abundance of different bacterial taxa inside a sample.

In our study group of 128 surgical oncological patients, intestinal microbiota composition was not significantly changed one week post cefuroxime treatment when compared to pretreatment condition for patients without mechanical bowel preparation, but some loss in taxonomic variety could be observed. Taken together, cefuroxime does not promote short -term dysbiosis in surgical patients without any additional perioperative procedures.

## Data availability statement

The datasets presented in this study can be found in online repositories. The names of the repository/repositories and accession number(s) can be found at: https://doi.org/10.5281/zenodo.7302945.

## Ethics statement

This study was reviewed and approved by the Clinical Research Ethics Committee of the Regional Institute of Oncology (IRO) Iasi. The patients/participants provided their written informed consent to participate in this study.

## Author contributions

Conceptualization and methodology, ICV-T, R-MA, ICI and M-GD. validation, ICI, M-GD. formal analysis, R-MA. investigation, ICV-T, ȘI, A-MM and E-RB. resources, ICI, M-GD. data curation, R-MA. writing—original draft preparation, ICV-T, R-MA. writing—review and editing, ICV-T, R-MA. visualization, R-MA. supervision, ICI and M-GD. project administration, M-GD. funding acquisition, M-GD and ICI. All authors contributed to the article and approved the submitted version.
