# Supplementary material for: The impact of cefuroxime prophylaxis on human intestinal microbiota in surgical oncological patients
Source: Front Microbiomes. 2023 Feb 2;1:1092771. doi: 10.3389/frmbi.2022.1092771 (PMC12993628; doi:10.3389/frmbi.2022.1092771)
Supplement: Supplementary file 3 [file DataSheet_3.docx]

Supplementary Material

The impact of cefuroxime prophylaxis on human intestinal microbiota in surgical oncological patients

**Irina Cezara Văcărean-Trandafir*^,^**^†^**, Roxana-Maria Amărandi^,^*^,^**^†^**, Iuliu Cristian Ivanov, Ştefan Iacob, Ana-Maria Muşină, Elena-Roxana Bărgăoanu and Mihail-Gabriel Dimofte**

*** Correspondence:** Irina Cezara Văcărean-Trandafir and Roxana-Maria Amărandi
[trandafirina.bi@gmail.com](mailto:trandafirina.bi@gmail.com) or [irina.trandafir@iroiasi.ro](mailto:irina.trandafir@iroiasi.ro) ; [rpomohaci@iroiasi.ro](mailto:rpomohaci@iroiasi.ro)

# Supplementary Figures


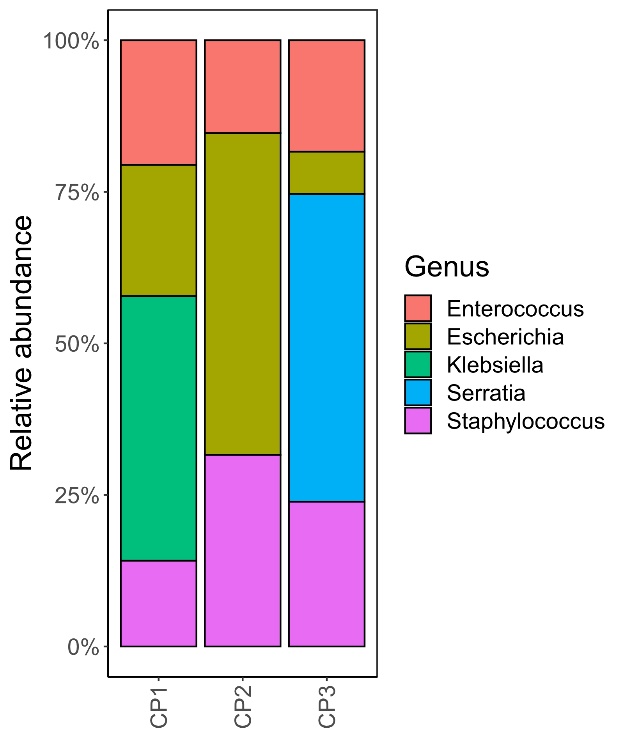


**Supplementary Figure 1.** Mock bacterial community structure merged at genus level per each type of positive control, in terms of relative abundances.


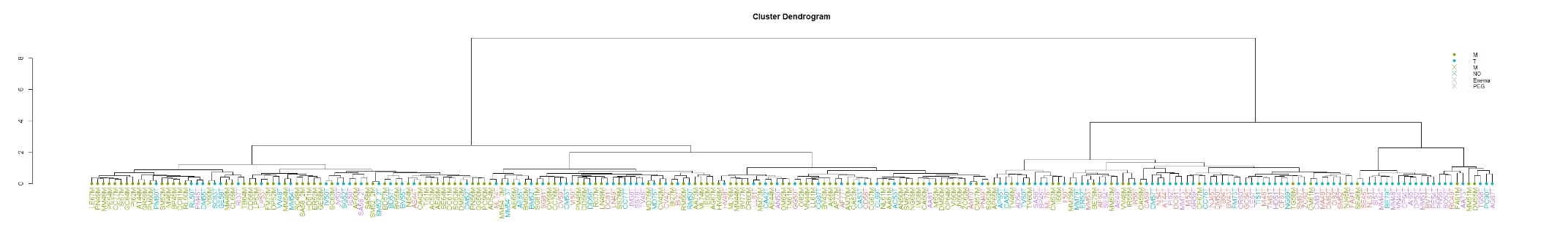


**Supplementary Figure 2.** Cluster dendrogram from hierarchical cluster analysis using Bray-Curtis dissimilarity and Ward’s clustering algorithm for VST-normalized abundance. Point color according to treatment type: samples before antibiotic treatment (M) – green; samples 7 days post-antibiotic treatment (T) – blue; Label color according to bowel cleansing preparation: green – samples before antibiotic treatment, with no bowel cleansing (M); blue – samples 7 days post-antibiotic treatment, with no bowel cleansing (NO); red – 7 days post-antibiotic treatment, with preoperative enema (Enema); purple – samples 7 days post-antibiotic treatment, with preoperative PEG MBP (PEG);
